# Mutation detection and inhibitor risk in Iranian patients with Hemophilia A: Six novel mutations

**DOI:** 10.1002/ccr3.3294

**Published:** 2020-09-15

**Authors:** Farzaneh Nasirnejad Sola, Saeid Morovvati, Mitra Sabetghadam Moghadam, Malihe Entezari

**Affiliations:** ^1^ Department of Genetics Faculty of Advanced Sciences and Technology Islamic Azad University of Medical Sciences Tehran Iran; ^2^ Human Genetic Research Center Baqiyatallah University of Medical Sciences Tehran Iran

**Keywords:** factor VIII gene, hemophilia A, inhibitor risk

## Abstract

This investigation facilitates a better understanding of inhibitor development, the critical treatment morbidity in HA patients. Furthermore, six novel mutations are reported, which would expand the mutation spectrum of the *F8* gene.

## Graphical abstract

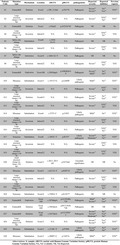

## 1 INTRODUCTION

Hemophilia A is known as a bleeding abnormality accompanied with recessive X‐linked inheritance. More than 3000 various mutations have been found in the *F8* gene. Intron 22 and Intron 1 inversions (Inv22 and Inv1) are the most frequent molecular variations observed in severe HA patients. The present study aimed to identify the *F8* gene mutations in Iranian patients with HA and examine their correlation with the development of inhibitors. This research was conducted on 30 Iranian HA patients, including 28 severe and two mild patients. IS‐PCR (inverse‐shifting PCR) was used to assay the inv22 and inv1 and to screen the other mutations, In Inv22, and Inv1‐negative patients, direct sequencing of whole 26 exons was performed by Sanger sequencing method. Finally, multiplex PCR was carried out to detect probable large deletions. Of all the detected mutations, 73.3% mutations (22 of 30) were classified as high‐risk mutations, including Inv22 in 12/30 patients (40%), frameshift mutations in 6/30 (23.3%), substitution nonsense in 2/30 (6.7%), and large deletions in 2/30 (6.7%). The analysis revealed six different mutations in the *F8* gene, which had not been previously reported based on different HA databases. Inhibitors were observed in 32% of patients with severe HA. No significant difference was found between the mutation risk groups and inhibitor development. We demonstrated that the distribution of the *F8* gene mutations and the prevalence of *F8* inhibitors in Iranian patients were consistent with those in different populations concerned in previous studies. The lack of correlation between mutation risk and inhibitor incidence can be attributed to the high prevalence of inhibitors in patients with splice site and missense mutations, as documented in some previous studies.

Hemophilia is considered as one of the most serious hemorrhagic disorders and is categorized into hemophilia A (HA), hemophilia B (HB), and hemophilia C (HC). There are three different coagulation factor deficiencies in these categories: factor VIII (FVIII) in HA, factor IX (FIX) in HB, and factor XI (FXI) in HC.^1^ HA (OMIM 306700) is defined as a bleeding abnormality with the X‐linked recessive inheritance that predominately affects males, with one in 5000 at birth. According to the level of coagulation FVIII, HA is classified into severe <1 IU/dL, moderate 1‐5 IU/dL, and mild 5‐40 IU/dL.^2^ There are several bleeding sites in this disease that vary from life‐threatening to non–life‐threatening. The fatal hemorrhages generally include organ bleeding, intracranial bleeding, gastrointestinal and genitourinary bleeding, and pharyngeal bleeding. Mucosal bleeding, bleeding into the joints (hemarthrosis), and muscles are classified as non–life‐threatening type. Hemarthrosis is one of the usual features of HA.^3^ FVIII, also known as antihemophilic factor (AHF), is one of the coagulation factors involved in the coagulation cascade. Activated FVIII (FVIIIa) that functions in the intrinsic tenase complex as the FIXa cofactor increases the catalytic efficiency of FIXa. Consequently, with activating FX, the pathway would convert prothrombin (FII) to thrombin (FIIa) and eventually fibrin to fibrinogen.^4^ Different mutations, including large rearrangements, intragenic deletions or insertions, and point mutations in the *F8* gene, have been found as the primary cause of the disease.^5^ Intron 22 (int22) and Intron 1 (int1) inversions (invs) are proved as the most common mutations in severe HA with the incidence rates of 45% and 5%, respectively.^6^ Int22 contains a CPG island that functions as a bidirectional promoter for genes known as factor VIII–associated A (F8A) and B (F8B). Two copies of F8A, also known as int22h‐1, are also located in the telomeric side of the gene, including int22h‐2 (proximal copy) and int22h‐3 (distal copy). There is also an identical copy of int1, known as int1‐2, upstream of the *F8* gene.^7^, ^8^ Homologous recombination between int1‐1 and int1‐2, int22h‐1 with one of its two copies, either int22h‐2 or int22h‐3, results in inv1 and inv22, respectively.^9^ Inv1 produces two‐hybrid transcripts, including the promoter and a part of exon 1 of the *F8* gene along with alternative exons of the *VBP1* gene, and exons of *BRCC3* gene, with alternative exons 2‐26 of the *F8* gene.^10^ Inv22, which divides the *F8* gene into two distinct regions (exons 1‐22 and exons 23‐26), produces defective coagulation FVIII due to the lack of a secretory polypeptide chain.^11^ Although inv22 type1 is more common than type 2, no clinical discrepancy is observed between the two groups of patients. To date, more than 3000 different mutations have been reported in *F8 *Gene Variant Databases such as several large deletions or insertions, point mutations including nonsense variations, missense mutations, and mRNA splicing site errors. Moreover, frameshift mutations caused by deletions or insertions may result in severe HA.^12^

Coagulation tests fail to identify carrier parents and their affected child in the prenatal diagnosis process. Hence, molecular detection has become a pivotal approach in prenatal diagnosis and also the genetic counseling of patients with a history of this disease.^13^, ^14^ Additionally, it is well‐known that specific *F8* mutations contribute to the development of FVIII inhibitors; hence, the detection of these mutations is essential to manage HA treatment.^15^

This study aimed to analyze the *F8* gene in Iranian HA patients and examine the correlation between the detected mutations and the inhibitor presence. The identified mutations were categorized into high‐risk (namely Inv22, Inv1, large deletions, nonsense, and frameshift mutations) and low‐risk (namely missense variants, in‐frame deletion/insertions, and splice site mutations) genotypes, consistent with Rodin's study suggesting an association between the *F8* genotype and inhibitor development.^16^

## 2 MATERIALS AND METHODS

### 2.1 Patients

This study was conducted on 30 HA patients referred to our Genetic laboratory of Iranian Hemophilia and Thrombophilia Association (MAHTA). Ethics approvals were gained from all of the patients. The patients’ age range varied from three months to 60 years. The clinical severity of the patients had been previously measured using standard 1‐stage clotting assay. A majority of patients had severe symptoms (28 severe and two mild patients). They manifested symptoms of the disease at an early age. Many of the severe patients suffered from hemorrhage, spontaneous mucosal bleeding, and bruising. Regarding the mild patients, symptoms were experienced at an older age. The FVIII inhibitor titers in all the patients were quantified using the Nijmegen modification of the Bethesda assay^17^ (Table 1).

**Table 1:** Clinical and demographic data of the patients

Patients code	Severity	Diagnosis time	Causes of diagnosis	Phenotype	Inhibitor
S1	F8 activity < 1% ( severe HA)	1 mo	Bleeding after circumcision	Hemarthrosis	No
S2	F8 activity < 1% ( severe HA)	6 mo	Bleeding after drawing blood	Hemarthrosis	Yes
S3	F8 activity < 1% ( severe HA)	1 mo	Bleeding after drawing blood	Hemarthrosis	Yes
S4	F8 activity < 1% ( severe HA)	2 mo	Bleeding after circumcision	Hemarthrosis	No
S5	F8 activity < 1% ( severe HA)	5 mo	Chest bruise	Mucosal bleeding	No
S6	F8 activity < 1% ( severe HA)	16 d	Bleeding after circumcision	Mucosal bleeding	Yes
S7	F8 activity < 1% ( severe HA)	1 mo	Bleeding after circumcision	Mucosal bleeding	Yes
S8	F8 activity < 1% ( severe HA)	1 d	Family history	Hemarthrosis	No
S9	F8 activity < 1% ( severe HA)	18 mo	Bleeding after drawing blood	Hemarthrosis	No
S10	F8 activity < 1% (severe HA)	1 mo	Bleeding after drawing blood	Mucosal bleeding	No
S11	F8 activity < 1% ( severe HA)	2 y	Eye bruise	Mucosal bleeding	No
S12	F8 activity < 1% (severe HA)	1 d	Family history	Hemarthrosis	Yes
S13	F8 activity < 1% (severe HA)	1 d	Family history	Hemarthrosis	No
S14	F8 activity < 1% ( severe HA)	1 d	Bleeding after circumcision	Hemarthrosis	No
S15	F8 activity < 1% ( severe HA)	1 d	Family history	Hemarthrosis	No
S16	F8 activity < 1% ( severe HA)	1 d	Bleeding after circumcision	Hemarthrosis	Yes
S17	F8 activity < 1% ( severe HA)	8 mo	Bleeding after circumcision	Mucosal bleeding	No
S18	F8 activity < 1% ( severe HA)	7 mo	Bleeding after circumcision	Hemarthrosis	No
S19	F8 activity < 1% ( severe HA)	2 y	Bleeding after circumcision	Mucosal bleeding	No
S20	F8 activity < 1% ( severe HA)	1 mo	bleeding after circumcision	Hemarthrosis	No
S21	F8 activity < 1% ( severe HA)	1 mo	Family history	Hemarthrosis	Yes
S22	F8 activity < 1% ( severe HA)	1 d	Bleeding after circumcision	Mucosal bleeding	No
S23	F8 activity < 1% ( severe HA)	1 d	Bleeding after circumcision	Hemarthrosis	Yes
S24	F8 activity < 1% ( severe HA)	6 mo	Body bruise	Mucosal bleeding	Yes
S25	F8 activity < 1% ( severe HA)	2 mo	Family history	Mucosal bleeding	No
S26	F8 activity < 1% ( severe HA)	1 d	Bleeding after circumcision	Mucosal bleeding	No
S27	F8 activity < 1% ( severe HA)	1 y	Bleeding after circumcision	Hemarthrosis	No
S28	F8 activity < 1% ( severe HA)	6 y	Testis bleeding	Hemarthrosis	No
S29	F8 activity 5%‐30% (mild HA)	23 y	aPTT test	‐	No
S30	F8 activity 5%‐30% (mild HA)	6 y	Hematoma in the knee	‐	No

Abbreviations: aPTT, activated partial thromboplastin time; S, sample.

### 2.2 Sample collection and DNA extraction

After obtaining informed consent from all the participants following the Declaration of Helsinki, peripheral blood samples were taken from the patients using EDTA tubes, and genomic DNA was extracted utilizing the standard salting‐out method.^18^

### 2.3 Detection of inversion mutations by IS‐PCR

The patients were first screened for the detection of Inv22 (type I and type II) and Inv1 rearrangements by the use of inverse‐shifting PCR (IS‐PCR) technique. We used the IS‐PCR protocol of Rossetti et al^9^ To summarize, genomic DNA was digested by BCL1 enzyme, and digested DNA fragments were separated. Then, DNA fragments were ligated with T4 DNA ligase and purified. Finally, PCR amplification was performed, and the products were run on a 2% agarose gel. The diagnosis was made based on different fragment lengths of the IS‐PCR products for the *F8* gene.

### 2.4 Sequencing the whole exons by Sanger sequencing method

The *F8* gene was directly sequenced for all 26 exons using the Sanger sequencing method. PCR was performed, and the products were then sequenced on ABI 3500 Genetic analyzer. The miscellaneous lines of in silico computational analysis (MutationTaster, CADD, PolyPhen, VarSome, SIFT, etc) were used to predict the pathogenicity of variations. These variants have also been investigated in HA databases (COSMIC, EAHAD, HGMD) to check previous reports.

### 2.5 Multiplex PCR

In two patients, when PCR was performed, some exons had not been amplified to be directly sequenced, suggesting a deletion affecting these exons. To determine the breakpoint regions, multiplex PCR was done for the associated exons, as described by Fernandez*‐*Lopez et al.^19^

### 2.6 Statistical analysis

Statistical significance was analyzed using SPSS software (IBM Corp. Released in 2019. IBM SPSS Statistics for Windows, Version 26; Armonk, NY: IBM Corp). The comparisons of the collected data were performed using Fisher's exact test. *P*‐value <.05 was considered to indicate statistical significance.

## 3 RESULTS

This study was performed on 30 Iranian patients with HA from unrelated families. The analysis of these patients revealed various types of mutations in the *F8* gene. Across the whole study group, the rate of mutation detection success was 100%. IS‐PCR revealed that 12 out of 30 samples (40%) were positive for Inv22 type1 with severe HA. Inv22 type 2 and inv1 were not found in any of the severe or mild patients.

Eighteen patients with no Inv22 mutation were then analyzed for other *F8* gene variations, and disease‐causing variants were detected in 16 patients (53.3%).

The mutations included six frameshifts, five missense substitutions, two nonsense substitutions, two substitutions in the splicing region, and one in‐frame deletion in distinct areas of the *F8* gene.

**Figure 1:**
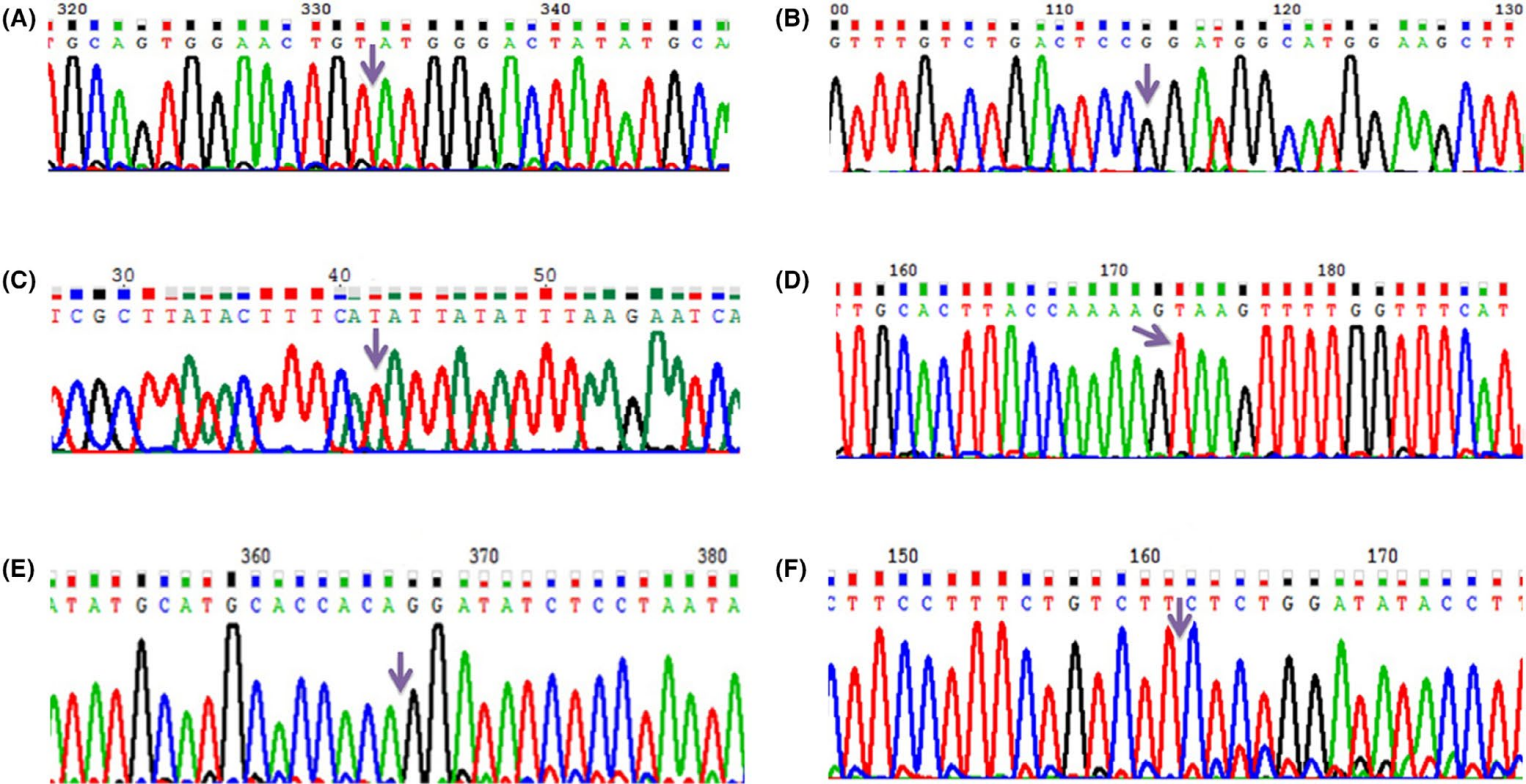
Sequences of novel mutations in F8 gene; (A) DNA sequence of exon 1 in sample 3, c.95delC p. S32Yfs*45; (B) DNA sequence of exon 8 in sample 5, c.1010‐2A>G; (C) DNA sequence of exon 10 in sample 7, c.1444‐1G>T; (D) DNA sequence of exon 16 in sample 24, c.5509A>T p.L1837*; (E) DNA sequence of exon 14D in sample 26, c.3943delA p.R1315Gfs*20; (F) DNA sequence of exon 13 in sample 20, c.2013_2015delCTT p.F672del

Nine different pathogenic, six likely pathogenic, and one uncertain significance variants were detected, among which six variants in the *F8* gene have not been previously reported in different databases, including HGMD, EAHAD, COSMIC, and VarSome. The six variants are two pathogenic deletions: c.95delC (p.Ser32TyrfsTer40) and c.3943delA (p.Arg1315GlyfsTer20) in exons 1 and 13, respectively, an uncertain significance deletion: c.2013_2015del (p.Phe672del) in exon 13, two pathogenic point mutations in splicing regions: c.1010‐2A>G and c.1444‐1G>T in exons 8 and 10, respectively; and one pathogenic nonsense mutation: c.5509A>T (P.Lys1837Ter) in exon 16 (Figure 1).

The detected c.2013_2015del mutation in the *F8* gene has not been previously reported for its pathogenicity. Based on the ACMG guidelines, this variant has been classified as a variant of uncertain significance (VUS); hence, direct sequencing was performed in some family members with or without the disease to confirm the mutation as a causing variant of the disease. The same mutation, c.2013_2015del (p.Phe672del), on the *F8* gene, was detected in a heterozygous state in the proband's mother and his affected maternal grandfather hemizygously.

According to multiplex PCR results, large deletions in the exons 8‐9 and exon 15 were identified in two other patients. Of all causative mutations identified in this study, 73.3% (22 of 30) were classified as high risk, including 12 invs, six frameshifts, two nonsense mutations, and two large deletions. Table 2 shows a list of all mutations.

**Table 2:** Genetic variants identified in 30 Iranian patients with HA

Patients Code	Mutation type	Mechanism	Location	cHGVS	pHGVS	Pathogenicity	Reported severity	Reported inhibitor	Previous reports
S1	Frameshift	Deletion	Exon 2	c.209_212del	p.Ffs*70	Pathogenic	Severe^36^ Severe^35^	No^36^ Yes^35^	Yes^35^
S2	Large structural change (>50 bp)	Inversion	Intron 22	N/A	N/A	Pathogenic	Severe^37^	Yes^37^ No^37^	Yes^37^
S3	Frameshift	Deletion	Exon 1	c.95delC	p.S32Yfs*45	Pathogenic	NR	NR	No
S4	Large structural change (>50 bp	Inversion	Intron 22	N/A	N/A	Pathogenic	Severe^37^	Yes^37^ No^37^	Yes
S5	Splice site change	Substitution	Exon 8	c.1010‐2A>G	N/A	Pathogenic	NR	NR	No
S6	Large structural change (>50 bp	Inversion	Intron 22	N/A	N/A	Pathogenic	Severe^37^	Yes^37^ No^37^	Yes
S7	Splice site change	Substitution	Exon 10	c.1444‐1G>T	N/A	Pathogenic	NR	NR	No
S8	Large structural change (>50 bp	Inversion	Intron 22	N/A	N/A	Pathogenic	Severe^37^	Yes^37^ No^37^	Yes
S9	Frameshift	Duplication	Exon 14B	c.2945dupA	p.N982Kfs*r9	Pathogenic	Severe^36^ Mild^38^	No^36^ No^38^	Yes^38^
S10	Missense	Substitution	Exon 13	c.1931T>G	p.L644W	Likely pathogenic	Mild^36^	No^36^	Yes^36^
S11	Large structural change (>50 bp	Inversion	Intron 22	N/A	N/A	Pathogenic	Severe^37^	Yes^37^ No^37^	Yes
S12	Large structural change (>50 bp)	Deletion	Exon 8‐9	N/A	N/A	Pathogenic	Severe^19^ Severe^36^	No^19^ Yes^36^	Yes^19^
S13	Large structural change (>50 bp	Inversion	Intron 22	N/A	N/A	Pathogenic	Severe^37^	Yes^37^ No^37^	Yes
S14	Missense	Substitution	Exon 4	c.575T>C	p.I192T	Likely pathogenic	Mild^36^	No^36^	Yes^36^
S15	Large structural change (>50 bp	Inversion	Intron 22	N/A	N/A	Pathogenic	Severe^37^	Yes^37^ No^37^	Yes
S16	Large structural change (>50 bp	Inversion	Intron 22	N/A	N/A	Pathogenic	Severe^37^	Yes^37^ No^37^	Yes
S17	Nonsense	Substitution	Exon 8	c.1063C>T	p.R355*	Likely pathogenic	Severe^40^ Moderate^39^	No^40^ No^39^	Yes^39^
S18	Large structural change (>50 bp	Inversion	Intron 22	N/A	N/A	Pathogenic	Severe^37^	Yes^37^ No^37^	Yes
S19	Large structural change (>50 bp	Inversion	Intron 22	N/A	N/A	Pathogenic	Severe^37^	Yes^37^ No^37^	Yes
S20	Small structural change (in‐frame, <50 bp)	Deletion	Exon 13	c.2013_2015del	p.F672del	Uncertain significance	NR	NR	No
S21	Missense	Substitution	Exon 21	c.6213A>T	p.R2071S	Likely pathogenic	Severe^41^	No^41^	Yes^41^
S22	Large structural change (>50 bp	Inversion	Intron 22	N/A	N/A	Pathogenic	Severe^37^	Yes^37^ No^37^	Yes
S23	Large structural change (>50 bp	Inversion	Intron 22	N/A	N/A	Pathogenic	Severe^37^	Yes^37^ No^37^	Yes
S24	Nonsense	Substitution	Exon 16	c.5509A>T	p.K1837*	Pathogenic	NR	NR	No
S25	Frameshift	Duplication	Exon 14D	c.3870dupA	p.G1291Rfs*29	Pathogenic	Mild^43^ Severe^42^	No^43^ Yes^42^	Yes^42^
S26	Frameshift	Deletion	Exon 14D	c.3943delA	p.R1315Gfs*20	Pathogenic	NR	NR	No
S27	Frameshift	Deletion	Exon 14D	c.3637delA	p.I1213Ffs*5	Pathogenic	Severe^45^ Severe^44^ Moderate^46^	No^45^ Yes^44^ No^46^	Yes^44^
S28	Large structural change (>50 bp)	Deletion	Exon 15	N/A	N/A	Pathogenic	Severe^19^ Severe^47^	Yes^19^ No^47^	Yes^19^
S29	Missense	Substitution	Exon 13	c.2110C>T	p.P704S	Likely pathogenic	Severe^48^ Mild^48^	No^48^ No^48^	Yes^48^
S30	Missense	Substitution	Exon 13	c.2090T>C	p.V697A	Likely pathogenic	Mildy^49^	No^49^	Yes^49^

Abbreviations: cHGVS, nucleic acid Human Genome Variation Society; NA, not available; NR, not reported; pHGVS, protein Human Genome Variation Society; S, sample.

Regarding the clinical severity of the 30 patients, 28 cases (93.3%) were severe, and two patients (6.6%) were mild ones.

The frequency of inhibitor positive patients was found to be 30% (9 out of 30). In patients with severe HA, large deletions, nonsense mutations, and splicing errors showed the highest incidence of inhibitors. The lowest inhibitor frequency in severe HA was found in patients with missense and frameshift mutations. Table 3 presents the details of the data.

**Table 3:** Incidence of inhibitors in patients with HA caused by different types of mutation

Mutation type	Inhibitor frequency (%)
Large deletion	1/2 (50)
Nonsense	1/2 (50)
Inversion 22	4/12 (33.3)
Frameshift	1/6 (16.7)
Splice site	1/2 (50)
Missense	1/5 (20)

In the high‐risk mutation group, inhibitor positivity was found in 7 out of 22 patients (32%). In the low‐risk mutation group, inhibitor development was detected in 2 out of 8 patients (25%). The association between the mutation risk group and inhibitor development was not statistically significant (*P* > .05). In the patients with Inv22, the frequency of inhibitor positivity was 33.3% (4 out of 12). The association between inv mutations and inhibitor development was not statistically significant (*P* > .05), as shown in Table 4.

**Table 4:** Inhibitor * inversion and mutation risk cross‐tabulation

		Inversion		*P*‐value	Risk		*P*‐value
		Negative	Positive		High	Low	
Inhibitor	Negative	13 (72.2%)	8 (66.7%)	.528	15 (68%)	6 (75%)	.547
	Positive	5 (27.8%)	4 (33.3%)		7 (32%)	2 (25%)	
Total	Count %of total	18 (100%)	12 (100%)		22 (100%)	8 (100%)	

Note

*P*‐value, probability value; significance level was set at *P* < .05.

## 4 DISCUSSION

The current study was conducted to investigate the *F8* gene mutations in 30 Iranian patients with HA. This research revealed different mutations in the *F8* gene, of which six have not been previously reported in the existing mutation register databases. Previous studies have reported inv22 and inv1 as the most common mutations in severe HA. Rossetti et al observed the presence of inv22 in 45%‐50% of severe patients.^9^ Moreover, in similar reports in Spain, Italy, Argentina, India, Egypt, Mexico, and Iran, inv22 is interpreted as the most frequent cause of severe HA.^20^, ^21^, ^22^ It should be noted that we reported the highest prevalence of inv22 mutation (40%), and this strongly supports previous studies. In most reports, the frequency of inv1 mutation has been stated to be 0%‐5% of patients with severe HA.^23^, ^24^ In our survey, none of the patients showed inv1 mutation. As expected, our findings confirm that invs are the most prevalent mutations in patients with severe HA; therefore, they should be examined as the first diagnostic step.

Missense errors are the most common mutations with a frequency of 70%‐80% among mild/moderate HA patients.^25^ We found missense mutations in both patients with mild HA (S29/S30). However, our findings are based on a limited number of mild patients. This research focused on patients with a severe phenotype, whereas it might be necessary to include more mild/moderate patients as well. It is probably because patients with severe HA are more likely to be medically concerned due to their more serious bleeding phenotype.

The FVIII mutation database consists of more than 3000 unique mutations in hemophiliac patients (EAHAD‐DB at dbs.eahad.org). We sequenced 18 patients with negative results for inv mutations and recognized six novel mutations not reported in any *F8* gene variant databases. Mutations c.95delC (p.S32Yfs*45), c.3943delA (p.R1315Gfs*20), and c.5509A>T (p.K1837*) are considered to be pathogenic as they introduce premature stop codons, resulting in protein truncation or loss altogether through mRNA nonsense‐mediated decay. Besides, we found two mutations in mRNA splicing region, c.1010‐2A>G and c.1444‐1G>T, in exons 8 and 10, respectively. The detected canonical splice site variants in the *F8* gene have not been previously reported for their pathogenicity. On the other hand, null variants, including canonical splice site errors affecting the *F8* gene, are a known mechanism of the disease, and multiple lines of in silico computational analysis (MutationTaster, CADD, etc) support the deleterious effect of the variants on the gene or gene product.

According to the data reported worldwide, severe HA patients have a higher prevalence of inhibitors than mild/moderate deficiency, as observed in 25%‐30% of severe patients and approximately 3%‐13% of patients with mild/moderate HA.^26^ In a study, the incidence of inhibitor development in 635 Iranian patients with severe HA was reported to be 22.8%.^27^ In another review study, 100 out of 355 patients (28%) from six main hemophilia care centers in Iran were positive for the FVIII inhibitor.^28^ Our study showed that 32% of patients with severe HA had factor VIII inhibitors. This finding was almost similar to those reported in previous studies.

In many investigations, the relationship between *F8* genotypes and inhibitor development varies noticeably; however, most reviews confirm that the inhibitor incidence is correlated with invs, large deletions, and nonsense mutations in severe HA.^29^ A survey in Germany showed a higher incidence of inhibitor in patients with large deletions, nonsense, and inv22 mutations, compared to patients with small deletions or insertions, missense, and splicing errors.^30^ In a large cohort of US patients with severe HA, the frequency rates of inhibitors were 57.1%, 35.7%, and 26.8% in large deletions, splice sites, and inv22, respectively.^31^ In another study, 1104 cases with HA from three different countries, including Iran, France, and the Netherlands, were analyzed. In this study, in contrast to previous studies, in patients with severe HA, splicing errors had the highest frequency of inhibitors, and the lowest inhibitor prevalence was observed in patients with missense mutations.^32^ Generally, in most studies, the inhibitor frequency ranges in severe HA patients are 21%‐27%, 25%‐40%, 10%‐16%, and 5%‐10% for inv22, nonsense mutations, frameshift mutations, and missense variations, respectively. For other genotypes, the results are more different, ranging from 22% to 67% and 17% to 50% for large deletions, and splice site changes, respectively.^31^ This study showed the equal inhibitor incidence in patients with large deletions, nonsense mutations, and splice site errors, which were higher than those in cases with inv22. Contrary to most of the previous studies, we observed a higher range of inhibitor frequency in patients with missense mutations and splice site changes. Hence, concerning the classification of mutations into low‐ and high‐risk groups, the incidence of inhibitor development in low‐risk mutations was close to those in the high‐risk group. In a study in Turkey, the examination of 270 patients with HA revealed a significant correlation between high‐risk genotypes and inhibitor formation. In our study, however, no significant difference was found between the mutation risk group and inhibitor development.^33^ The lack of correlation in the present study can be attributed to the high prevalence of inhibitors in patients with splice errors, as documented in one previous study. Furthermore, missense variations are associated with an overall lower risk of inhibitor development; however, different missense mutations are correlated with specific risks for inhibitor formation. For example, the likelihood of inhibitor formation in association with the *F8* missense mutations is significantly higher if the amino acid substitution is related to another physicochemical class than the original residue. Another possible reason for this is that we cannot rule out that positive family history of inhibitors, ethnicity, and other genetic factors, including certain polymorphisms in immune‐modulatory genes, might have influenced the risk of inhibitor development. Interestingly, *F8* genotypes are believed to be equally distributed across different ethnic populations; however, this may not be true for other genetic risk factors.^34^

One of the limitations in this research was that the surveys included a small number of patients; nevertheless, the assessment of the inhibitor risk caused by genetic factors would require the collection of data on much larger numbers of patients using a population‐based approach. Despite this, we can still claim a close agreement with the findings of previous publications in many aspects, including the prevalence of mutations, overall inhibitor incidence, and its distribution in each of the mutations, and this confirms that our findings are appreciable. We recommend that further studies such as meta‐analysis studies can yield more precise estimates of the inhibitor risk for different types of *F8* mutations.

## 5 CONCLUSION

A high mutation detection rate was achieved using the broad molecular techniques performed in this study, which included six novel mutations. The results provided additional support for the distribution rate of *F8* gene mutations. A high range of inhibitor frequency was observed in patients with missense and splice site mutations. The prevalence of *F8* inhibitors was close to those observed in previous reviews. However, the relationship between *F8* genotypes and inhibitor development was not significant.

## CONFLICT OF INTEREST

None declared.

## AUTHOR CONTRIBUTIONS

FNS: involved in implementation of research and obtained funds. SM: involved in clinical evaluation and diagnosis. MSM: is author of the concept and provided objectives of the paper, collected the data, analyzed and interpreted the data, involved in statistical analysis, prepared the manuscript and approved the final version of the manuscript. ME: worked out the literature.

## CONSENT STATEMENT

Published with written consent of the patient.
